# A confirmed feeding attempt by the haematophagous horse fly Philoliche (Philoliche) rondani (Bertoloni, 1861) (Diptera: Tabanidae) on fresh carrion

**DOI:** 10.3897/BDJ.10.e77507

**Published:** 2022-03-31

**Authors:** Benjamin Miller, Martin Villet, John Mark Midgley

**Affiliations:** 1 Rhodes University, Makhanda, South Africa Rhodes University Makhanda South Africa; 2 Southern African Forensic Entomology Research Laboratory, Makhanda, South Africa Southern African Forensic Entomology Research Laboratory Makhanda South Africa; 3 KwaZulu-Natal Museum, Pietermaritzburg, South Africa KwaZulu-Natal Museum Pietermaritzburg South Africa

**Keywords:** feeding evolution, anticoagulant, forensic entomology, haematophagous insects

## Abstract

**Background:**

Many flies have specially evolved feeding mechanisms to imbibe liquids of specific viscosities. Observations of feeding on atypical liquids are notable because of their rarity.

**New information:**

We report the first record of intrusive fluid feeding on vertebrate carrion by *Philolicherondani*.

## Introduction

Diptera constitute one of the five taxonomically megadiverse insect orders (Costello et al. 2013) and embody considerable functional diversity, exemplified by the feeding habits of adult flies. Many flies are generalist feeders, but specialised feeding has evolved independently in several dipteran lineages (Krenn et al. 2005). Feeding specialisation is typically responsive to liquid foods, but within this dietary category, there is morphological diversity related to the nature of the target liquids (Karolyi et al. 2014). The viscosity and homogeneity of liquids play important roles in shaping the evolution of feeding mechanisms (Rotheray 2012, Kim et al. 2013).

Generalist liquid feeders, such as the Muscidae, have short mouthparts and consume food by capillary action with sponge-like mouthparts. Specialised feeders have modified mouthparts to access food resources more efficiently, such as long-tongued nectar feeders or to access otherwise inaccessible food, such as heamatophages. Haematophagous tabanids are telmophagous, feeding on blood that pools at the site where their mouthparts have formed a laceration. However, all haematophages must overcome their hosts' haemostatic responses to retrieve a blood meal (Ribeiro 1987). This usually requires a form of anticoagulant, produced from the salivary glands, to be secreted into the wound to prevent blood clotting. Amongst the Tabanidae, a diversity of compounds exhibit such properties, acting to prevent platelet aggregation, cause vasodilation or inhibit coagulation. These salivary gland compounds are often species-specific in their mode of action and composition and highly varied. However, thrombin and factor Xa inhibitors appear most common across the haematophagous Diptera (Kazimírová et al. 2002a, Kazimírová et al. 2002b). While the prevention of coagulation has been studied, it is unclear what effects these compounds have on the reversal of this process.

Specialised vertebrate blood-feeding has evolved multiple times in the Diptera and is observed in four of the five major divisions; mammals, birds, reptiles and amphibians (Gibson and Torr 1999, Krenn et al. 2005). All terrestrial vertebrate groups are prey. Blood is a viscous emulsion rather than a true liquid and haematophagous insects require specialised feeding apparatus for the uptake of undigested blood. Generalists can take up blood that has been broken down either through extra-oral or bacterial digestion (Sze et al. 2012, Rivers et al. 2014). Given the difficulty presented by the uptake of viscous fluids, atypical feeding by specialised animals is noteworthy. Atypical feeding is also of interest in forensic entomology. Feeding marks created by animals can link corpses to specific habitats or outdoor locations and provide evidence of post mortem movement (e.g.Backwell et al. 2012, de Souza et al. 2020).

## Taxon treatments

### Philoliche (Philoliche) rondani

(Bertoloni, 1861)

19FAFF98-8D86-5F61-BD1E-029FBC8D2480

#### Materials

- **Type status:** Other material. **Occurrence:** individualCount: 1; sex: female; lifeStage: adult; behavior: feeding; occurrenceStatus: present; preparations: photograph; **Taxon:** scientificName: *Philolicherondani*; acceptedNameUsage: *Philolicherondani*; parentNameUsage: Tabanidae; kingdom: Animalia; phylum: Arthropoda; class: Insecta; order: Diptera; family: Tabanidae; genus: Philoliche; specificEpithet: *rondani*; taxonRank: species; scientificNameAuthorship: (Bertoloni, 1861); nomenclaturalCode: ICZN; taxonomicStatus: accepted; **Location:** higherGeographyID: 7017573; higherGeography: Africa: South Africa: Limpopo: Mookgophong: ASDIA Wild Game Farm; continent: Africa; country: South Africa; countryCode: ZA; stateProvince: Limpopo; locality: ASDIA Wild Game Farm; verbatimLocality: ASDIA Wild Game Farm, Mookgophong region; locationAccordingTo: Getty Thesaurus of Geographic Names; verbatimCoordinates: 24°26'S 28°25'E; verbatimLatitude: 24°26'S; verbatimLongitude: 28°25'E; verbatimCoordinateSystem: degrees minutes; decimalLatitude: -24.43333; decimalLongitude: 28.41666; **Identification:** identifiedBy: John Chainey; **Event:** eventDate: 2014-12; startDayOfYear: 334; endDayOfYear: 365; year: 2014; month: 12; verbatimEventDate: December 2014; habitat: savanna; eventRemarks: about 18 hours post mortem; **Record Level:** type: StillImage; modified: 2014-12; rights: Content licensed under Creative Commons Attribution 4.0 International; rightsHolder: R Boon; basisOfRecord: HumanObservation

#### Ecology

**Figure 1.**
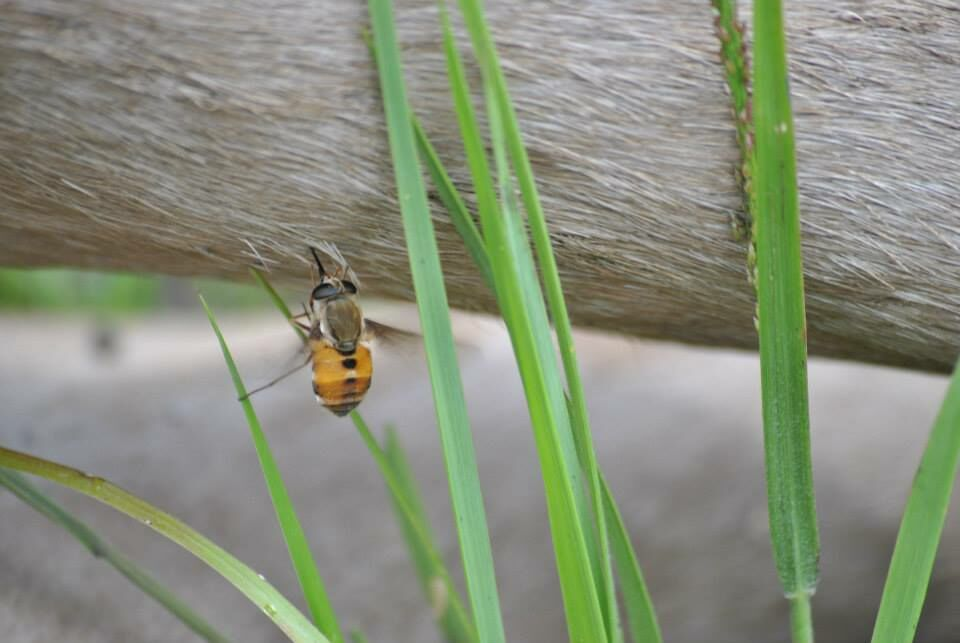
A female of Philoliche (Philoliche) rondani (Bertoloni, 1861) feeding on the carcass of a blue wildebeest (*Connochaetestaurinus* (Burchell, 1823)) that died the previous day, approximately 18 hours earlier (Photograph by R. Boon, with permission).

At the ASDIA Wild Game Farm (24°26'S, 28°25'E) in the Mookgophong area of Limpopo, South Africa, a female of Philoliche (Philoliche) rondani (Bertoloni, 1861) was photographed visiting the carcass of a blue wildebeest (*Connochaetestaurinus* (Burchell, 1823)) cow that had died during calving (Fig. 1). An additional specimen was observed, but not photographed. No specimens were collected. The incident occurred during December 2014 (austral summer). The flies' activity was located primarily on the lower part of the soft abdominal region of the carcass, closer to the ground. The flies were observed on the morning following the death, about 18 h post mortem. Accurate meteorological data is not available, but typical weather for December is warm, average minumum temperature is 17.4°C and average maximum 22.8°C. The flies were active when the observers arrived at the carcass and continued to visit the carcass for approximately five minutes. There was no obvious evidence of the animal being fed on by mammalian scavengers during the night, except that the calf had been dragged away from the remains of the mother. Adult blow flies already had a strong presence on other parts of the carcass.

#### Notes

A random selection of P. (P.) rondani specimens was taken from the KwaZulu-Natal Museum entomology collection (n = 15) and the average ratio of proboscis length (oral margin to tip of proboscis) to head height (vertex to oral margin) was measured using vernier calipers.

Measurements of preserved specimens found the ratio of proboscis to head height to be 1.59:1 (n = 15). The minimum observed ratio was 1.15:1. In Fig. 1, the ratio of visible length of the proboscis to head height is 1.10:1, indicating that the labellae have penetrated into the tissue to obtain a blood meal. This represents the first record of intrusive feeding on a post mortem host by Tabanidae.

## Discussion

The average night time temperature in Mookgopong in December is 17.4°C, which is not low enough to delay decomposition or blood coagulation. Given the depth that the proboscis has penetrated and the time since death, intrusive feeding on coagulated blood is the most likely explanation for this observation. Further investigation into the oral secretions of P. (P.) rondani is needed to establish the ability of these secretions to reverse the coagulation of blood. The reversal of coagulation is a likely explanation for this feeding observation, given the morphological specialisation in *Philoliche*.

Several authors have noted Tabanidae at carcasses, but these observations have largely been dismissed as incidental (Oldroyd 1957, Payne and Crossley 1966, Martinez et al. 2007, Villet 2017, Cusser et al. 2021). This may seem logical, as coagulating blood may be too viscous to be consumed in the normal manner but, in light of this observation of intrusive feeding, these interactions might better be described as rare, rather than incidental. These observations have been made in the Nearctic (two), Afrotropical (two) and Neotropical (one) realms and from the subfamilies Tabaninae (three), Pangoniinae (one) and Scepsidinae (one). The variety in distribution and diversity of these observations indicates that more attention needs to be given to potential carcass feeding in Tabanidae and the potential range of viscosity in their food.

This behaviour is relevant to the field of forensic entomology and further investigation into the frequency of this behaviour is warranted. In future, known post-mortem bite marks should be documented, as the physiological response is likely to be different from that in pre-mortem bites. Post-mortem bite marks indicating feeding by Tabanidae provide evidence for their presence on a corpse and could show that a corpse has been moved after death (Villet 2017).

## Supplementary Material

XML Treatment for Philoliche (Philoliche) rondani
